# Organisation: Voluntary, or Compelled?

**Published:** 1918-03-23

**Authors:** 


### ORGANISATION: VOLUNTARY, OR COMPELLED?

Organisation has just now such tremendous triumphs to its credit that there is little wonder the term is regarded as the master-word of the moment. Its successes are displayed on a large and public scale, and causes and interests having the widest possible claim depend upon its efficient application. To speak of it as an advantage is inadequate; rather it is a necessity of national existence. Even to hint that together with its gains there may be penalties will hardly be popular, and such penalties as exist must certainly be accepted. Individuals may suffer and injustices may be inflicted, but these considerations do not weaken the wider demand. Broadly this position is accepted, and it is accepted not only as a necessity for the moment, but as a guide for the future. It cannot be doubted that in the large and varied enterprises which come under the term "reconstruction" the principle of organisation has already gained a commanding position.

Like other activities of national importance, medicine will have to reckon with this new demand. Moreover, there are intrinsic reasons which point to the same conclusion. The growing complexity of medical investigation and the multiplication of highly technical methods of diagnosis and treatment are compelling a recognition of the need for co-operation and co-ordination. Thus from within, as well as from without, is coming the message that the day of individual and detached activity is in large measure over. The public good demands some scheme of organisation, and unless this is evolved from within it is almost certain to be imposed from without. The issue is without question one of the most serious which the profession can be called upon to face, and it is not likely to brook delay. There is good ground for believing that official and other activities are already at work beneath the surface, and it is more than probable that among these are ambitions which bode anything but good to the freedom of the profession. It is in these circumstances that is displayed the banner of a State Medical Service, and the phrase has an engaging quality to many minds. Whether all those who use the term mean one and the same thing may be doubted, and equally it may be doubted whether some of its patrons use it with any precise meaning. But there the term is, and it is not without its portent. Behind it there certainly lies the conviction that medical work must be arranged in some scheme of organisation, and this principle, as we have already allowed, hardly awakens a challenge. To discuss the phrase without some precise definition seems to us a mere waste of time, and we have no inclination to such a task. What is important is that the profession should recognise that something which this name may cover has to be faced and there is need to consider how the position so presented can be met. That there exists any general desire for official and bureaucratic control is hard to believe at a time when the bungling and expense of Government management is in all our ears. Unlimited competition and individual choice may have their disadvantages, but they do not obstruct personal freedom and initiative, and when they fail the blunderer pays his own penalty. Allow that they are just now largely out of fashion, there is still a wide difference between organisation which is self-imposed and organisation imposed by an official machine. Between these two, it seems to us, the profession will shortly have to make a choice, and if the capacity for organisation is not forthcoming within its own borders, it is likely that the heavy and unsympathetic hand of legislative compulsion will seize the situation.

